# AGE-FRIENDLY HEALTHCARE: AN EVOLUTIONARY CONCEPT ANALYSIS

**DOI:** 10.1093/geroni/igae098.3783

**Published:** 2024-12-31

**Authors:** Anna Zisberg, Paule Sarah Fraiman, Nosaiba Rayan-Gharra, Alexandra Danial-Saad, Dikla Segel-Karpas, Sharon Ost-Mor, Shoshi Keisari

**Affiliations:** University of Haifa, Haifa, Hefa, Israel; University of Haifa, Haifa, Hefa, Israel; University of Haifa, Haifa, Hefa, Israel; University of Haifa, Haifa, Hefa, Israel; University of Haifa, Haifa, Hefa, Israel; University of Haifa, Haifa, Hefa, Israel; University of Haifa, Haifa, Hefa, Israel

## Abstract

Current global aging trends emphasize the need to adapt healthcare systems to address older adults’ specific needs. Numerous age-friendly healthcare initiatives and approaches have emerged, but the field lacks a unified framework. The current study used Rodger’s evolutionary concept analysis model to explore ‘age-friendly healthcare’, examining the concept’s characteristics, components and structure. An initial literature search in multiple databases to identify articles relevant to age-friendly healthcare retrieved 1,407 articles. After screening for duplicates and relevance, 140 articles were examined for eligibility based on inclusion criteria related to age-friendly care, language, and full-text availability. Following full-text screening, 65 articles were included for data extraction by multiple researchers to synthesize theoretical, methodological and design elements. We identified similar terms, related cases, antecedents and consequences of age-friendly healthcare, as well as distinguishing attributes: Respect for older adults’ autonomy and needs; Leadership and organizational knowledge and support; Proactive policies and processes of care; Holistic care environments; and Communication and follow-up with awareness of challenges and barriers as well as prioritization of continuity-of-care. An overarching definition was crafted based on these findings. The concept of age-friendly healthcare is still in development, with much research focused on development and implementation but not yet on real-world patient and health-system outcomes. However, our proposed definition and analysis of the concept may help unify the field, clarify future research directions, and identify areas requiring further study. It may also enable development of improved practices and policies for the implementation of age-friendly healthcare in a variety of settings.

